# *BmREEPa* Is a Novel Gene that Facilitates BmNPV Entry into Silkworm Cells

**DOI:** 10.1371/journal.pone.0144575

**Published:** 2015-12-14

**Authors:** Xiao-long Dong, Tai-hang Liu, Wei Wang, Cai-xia Pan, Yun-fei Wu, Guo-yu Du, Peng Chen, Cheng Lu, Min-hui Pan

**Affiliations:** 1 State Key Laboratory of Silkworm Genome Biology, Southwest University, Chongqing, China; 2 Key Laboratory for Sericulture Functional Genomics and Biotechnology of Agricultural Ministry, Southwest University, Chongqing, China; Institute of Plant Physiology and Ecology, CHINA

## Abstract

We previously established two silkworm cell lines, BmN-SWU1 and BmN-SWU2, from *Bombyx mori* ovaries. BmN-SWU1 cells are susceptible while BmN-SWU2 cells are highly resistant to BmNPV infection. Interestingly, we found that the entry of BmNPV into BmN-SWU2 cells was largely inhibited. To explore the mechanism of this inhibition, in this study we used isobaric tags for relative and absolute quantitation (iTRAQ)-based quantitative protein expression profiling and identified 629 differentially expressed proteins between the two cell lines. Among them, we identified a new membrane protein termed BmREEPa. The gene encoding BmREEPa transcribes two splice variants; a 573 bp long *BmREEPa-L* encoding a protein with 190 amino acids and a 501 bp long *BmREEPa-S* encoding a protein with 166 amino acids. BmREEPa contains a conserved TB2/DP, HVA22 domain and three transmembrane domains. It is localized in the plasma membrane with a cytoplasmic C-terminus and an extracellular N-terminus. We found that limiting the expression of *BmREEPa* in BmN-SWU1 cells inhibited BmNPV entry, whereas over-expression of *BmREEPa* in BmN-SWU2 cells promoted BmNPV entry. Our results also indicated that BmREEPa can interact with GP64, which is the key envelope fusion protein for BmNPV entry. Taken together, the findings of our study revealed that BmREEPa is required for BmNPV to gain entry into silkworm cells, and may provide insights for the identification of BmNPV receptors.

## Introduction

The baculovirus, *Bombyx mori* nucleopolyhedrovirus (BmNPV) is a major pathogen of silkworm, which is an economically important insect and lepidopteran model. Its infection leads to about 70% annual loss in sericulture [1,2]. Because the mechanism of BmNPV resistance in silkworm is unclear, silkworm strains with high resistance to BmNPV infections have not been established. Therefore, the mechanism underlying BmNPV resistance in silkworm has become a subject of intensive investigation. Previous studies attempted to explore this resistance mechanism mainly using transcriptomes or proteasome analysis. Zhou et al. compared the transcriptomes of two silkworm lines that differ in their resistance to BmNPV and identified several differentially expressed genes including amino acid transporters, serine proteases and serpins [3]. They also found that proteasome can inhibit viral proliferation effectively [3]. Sagisaka et al. compared the transcriptome from the silkworm ovary cell lines pre- and post-BmNPV infection and found that the expression of *BmEts*, *BmToll10-3*, *tetraspanin*, *MMPvaliant1* and *ABC transporter* was increased while the expressions of *HSP20* and *HSP90* were reduced by BmNPV infection [4]. Using proteomic analysis Qin et al. found that caspase-1 and serine protease may also be related to antiviral activities [5]. Furthermore, Salem et al. analyzed the transcriptome of Sf21 cells pre- and post- AcMNPV infection and found that HSP70, HSC70 and some uncharacterized proteins play important roles in AcMNPV infection [6]. In addition, *lipase*, *BmNOX* and *Actin 3* were also shown to have antiviral activity [7,8]. Although a number of such studies have identified the viral infection-related host genes in recent years, a receptor for BmNPV has not been reported yet.

Receptor expression-enhancing protein (REEP) family is a gene family that can enhance receptor function. It was first identified because of its function in increasing the expression of ORs (olfactory receptor) and a number of GPCRs (G protein-coupled receptors) [9,10]. REEPs contain a TB2/DP1, HVA22 domain involved in the cellular transport and secretion [9]. This family consists of six members namely REEP1-REEP6, and can be divided into two subfamilies REEP1-REEP4 and REEP5-REEP6. There are generally 5 to 6 REEP genes in vertebrates, and two REEP genes in invertebrates [11–13] with each member responsible for different functions: REEP1 has been related to hereditary spastic paraplegia (HSP) protein, REEP2 can enhance the function of sweet taste receptors, REEP3 has been identified as a candidate gene for autism, REEP4 plays an important role in muscle and neural development, REEP5 positively correlates with major depression disorder (MDD), and REEP6 knockout causes necrosis of mouse retinal cells and lethality in zebrafish embryos [9–12,14–18]. However, the function of REEP genes in invertebrates especially in insects is unclear.

Previously, we established two cells lines, BmN-SWU1 and BmN-SWU2, from the silkworm ovary. These two cell lines significantly differ in their susceptibility to BmNPV infection: BmN-SWU1 is susceptible to infection while BmN-SWU2 is highly resistant to the BmNPV [19]. Our previous studies showed that the high resistance of BmN-SWU2 to BmNPV infection is due to the limited viral entry and suppressed viral DNA replication in these cells [20]. Therefore, we reasoned that these two cell lines were ideal for the identification of the BmNPV receptor and for the study of insect antiviral mechanisms. To understand the mechanisms regulating the differences in BmNPV resistance in these two cell lines, we used iTRAQ-based proteomic technology and determined the differential expression pattern of proteins in the two cell lines. The results led to the characterization of a membrane protein termed, BmREEPa, which may regulate BmNPV entry into silkworm cells.

## Materials and Methods

### Tissues, Cell Lines and Viruses

All silkworm materials used in our study were isolated from the P50 strain, which is preserved in the Silkworm Gene Bank at the Southwest University, Chongqing, China. Two ovarian cell lines, BmN-SWU1 and BmN-SWU2, were established from the ovarian tissue of 3-day-old 4^th^ instar *B*. *mori* larvae of the 21-872nlw strain [19]. In this study, v39K^prm^-eGFP and vHSP70^prm^-eGFP BVs (budded virus) were used. The recombinant BmNPV v39K^prm^-eGFP and vHSP70^prm^-eGFP constructs containing an EGFP reporter gene regulated by the virus inducible promoter, 39K (provisional Chinese Patent No. 201010231957.9, Pan et al.) and insect heat shock protein promoter, HSP70 (*Drosophila*; provided by Dr. Ai-chun Zhao, Sounthwest University), respectively were generated using the Bac-to-Bac Baculovirus Expression System (Invitrogen, Carlsbad, CA, USA) as previously described [21, 22]. The constructs were stored in dark at 4°C in TC100 insect medium.

### Protein Preparation and Proteomics Analysis

The BmN-SWU1 and BmN-SWU2 cells were culture at 27°C in TC100 medium (US Biological, Swampscott, MA, USA) supplemented with 10% heat-inactivated fetal bovine serum (FBS, GE Healthcare, Piscataway, NJ, USA). Protein extraction, isobaric tag for relative and absolute quantitation (iTRAQ) Labeling and SCX fractionation of proteins and LC-ESI-MS/MS analysis were conducted in BGI (Beijing, China). All experiments were performed twice.

For protein extraction, 10^7^ cells from each cell line were used, and 100 μg total proteins from each sample were digested with Trypsin Gold. Then, the peptides were dried and labeled with the iTRAQ tags. SCX chromatography was performed with a LC-20AB HPLC Pump system (Shimadzu, Kyoto, Japan). The iTRAQ-labeled peptide mixtures were reconstituted with 4 mL buffer A (25 mM NaH_2_PO_4_ in 25% ACN, pH 2.7) and loaded onto a 4.6 × 250 mm Ultremex SCX column containing 5 μm particles (Phenomenex). The peptides were eluted at a flow rate of 1 mL/min with a gradient of buffer A for 10 min, 5%−60% buffer B (25 mM NaH_2_PO_4_, 1 M KCl in 25% ACN, pH 2.7) for 27 min, and 60%−100% buffer B for 1 min. The system was then maintained at 100% buffer B for 1 min before equilibrating with buffer A for 10 min prior to the next injection. Elution was monitored by measuring the absorbance at 214 nm, and fractions were collected every 1 min. The eluted peptides were pooled as 20 fractions, desalted with a Strata X C18 column (Phenomenex) and vacuum-dried.

Each fraction was resuspended in buffer A (2% ACN, 0.1%FA) and centrifuged at 20000 g for 10 min. The final average peptide concentration was 0.5 μg/μl. Then, 10 μl of the supernatant was loaded on a LC-20AD nanoHPLC (Shimadzu, Kyoto, Japan) by the autosampler onto a 2 cm C18 trap column. The peptides were eluted in a 10 cm analytical C18 column (inner diameter 75 μm) packed in-house. The samples were loaded at 8 μl/min for 4 min, then the 44 min gradient was run at 300 nl/min starting from 2 to 35% B (98%ACN, 0.1%FA), followed by 2 min linear gradient to 80%, and maintained at 80% B for 4 min, and finally returned to 5% for 1 min. The peptides were subjected to nanoelectrospray ionization followed by tandem mass spectrometry (MS/MS) in an QEXACTIVE (Thermo Fisher Scientific, San Jose, CA) coupled online to a HPLC. Intact peptides were detected in the Orbitrap at a resolution of 70000. Peptides were selected for MS/MS using high-energy collision dissociation (HCD) operating mode with a normalized collision energy setting of 27.0; ion fragments were detected in the Orbitrap at a resolution of 17500. A data-dependent procedure that was altered between one MS scan followed by 15 MS/MS scans was applied for the 15 most abundant precursor ions above a threshold ion count of 20000 in the MS survey scan followed by Dynamic Exclusion duration for 15 s. The electrospray voltage applied was 1.6 kV. Automatic gain control (AGC) was used to optimize the spectra generated by the orbitrap. The AGC target for full MS was 3e6 and for MS2 it was 1e5. For MS scans, the m/z scan range was 350 to 2000 Da. For MS2 scans, the m/z scan range was 100−1800.

### Data Analysis

Proteins exhibiting more than 1.5 fold in their expression levels were considered to be differentially expressed. The Gene ontology (GO) classification IDs of the differentially expressed proteins were obtained from SilkDB (ftp://silkdb.org/pub/current/otherdata/Gene_ontology/silkworm_glean_gene.go) using Perl language. GO classification was performed with WEGO (http://wego.genomics.org.cn/cgi-bin/wego/index.pl) [3]. Sequences of the differentially expressed proteins were obtained from the Silkworm Genome Database (http://www.silkdb.org/silkdb/doc/download.html) using Perl. Kyoto Encyclopedia of Genes and Genomes (KEGG) pathways of the differentially expressed genes were annotated using the KAAS database (http://www.genome.jp/kaas-bin/kaas_main?mode=partial) and the KEGG pathways were classified based on the KEGG database (http://www.genome.jp/kegg/pathway.html) [3].

### Quantitative RT-PCR (qRT-PCR)

Total RNAs from BmN-SWU1 and BmN-SWU2 cells were extracted separately using E.Z.N.A.Total RNA Kit I (Omega, Norcross, GA, USA) and transcribed as previously described [23]. The silkworm ribosomal protein gene, *rpl3*, was used as the internal control. qRT-PCR was performed using a CFX96^TM^ Real-time system (Bio-Rad, Carlsbad, CA, USA). Amplification was carried out in a 15 μl reaction volume containing 1 μl of cDNA, 0.5 mM of each primer and 1× iTaq^TM^ Universal SYBR Green Supermix (Bio-Rad) in each well of a 96-well plate. The reaction conditions were as follows: 94°C for 30 s, followed by 40 cycles of denaturation at 95°C for 5 s and annealing and extension at 60°C for 15 s. Finally, the melt curve analysis was performed from 65°C to 95°C with 0.5°C increment for 5 s in each step.

### Viral Infection Assay

To determine infection efficiency of BmNPV, silkworm cells were infected with v39K^prm^-eGFP BVs at a multiplicity of infection (MOI) of 2 for 48 h at 27°C. Infected cells were observed through a fluorescence microscope (Olympus, Tokyo, Japan), washed three times in Phosphate Buffered Saline (PBS), suspended in TRIzol reagent (Invitrogen) for total RNA extraction and qRT-PCR to determine *VP39* expression level. The viral titer was determined from the culture fluid of infected cells using the 50% tissue culture infectious dose (TCID50) analysis. Each assay was repeated three times.

### Viral Titer Assay

v39K^prm^-eGFP BVs was serially diluted 10-fold from 10^−1^ to 10^−10^ and 100 μl of each dilution was inoculated in 96 well microtiter plates with each dilution in 8 wells. Then, 100 μl of the silkworm cell suspension (3 × 10^5^ cells/ml) was added per well while the controls received PBS. The numbers of eGFP positive wells were recorded every day for 5 days to calculate the viral titer using TCID50 analysis. Then, TCID50 value was converted into plaque forming units (PFUs). Each assay was repeated three times.

### Construction of RNAi and Over-Expression Vectors

We synthesized the miRNA (GenScript, Nanjing, China) and constructed the BmREEPa-RNAi vector as previously described [21]. BmREEPa-L, BmREEPa-S, BmREEPa-N terminus with Flag and BmREEPa-C terminus with Flag were cloned and ligated into pIZ-DsRed-V5/his vectors using vector specific primers (Table 1) and restriction endonucleases EcoRI and SacII (Takara, Dalian, China). BmREEPa with Flag tags and GP64 were cloned and ligated into pIZ-V5/his vectors using vector specific primers (Table 1) and restriction endonucleases, EcoRI and SacII (Takara).

**Table 1:** Primers used in this study.

Primer	Sequences
**BmREEPa-TA_F**	TCACATTCGGTTAGCCACTAAAAG
**BmREEPa-TA_R**	CGCATAAGCGAAGTGCTGAAA
**pIZ-BmREEPa_F**	cgGAATTCgATGGCATCCAAATTACAAGAGT
**pIZ-BmREEPa-S_R**	ccgCTCGAGcgATCTTGTTTCCCTGTGTTGGCC
**pIZ-BmREEPa-L_R**	ccgCTCGAGcgCAGCTTTTTAAGTGTCTTGCTGA
**pIZ-GP64_F**	cgGAATTCgcATGGTAGGCGCTATTGTTTTA
**pIZ-GP64_R**	ccCTCGAGcaTATTGTCTACTATTACGGTT
**Q-RT-BmREEPa_F**	GTCTATGAAGGCTCTGGAGTC
**Q-RT-BmREEPa_R**	TAAGGACGAATGATACGGTAGTAG
**Q-RT-VP39_F**	CTAATGCCCGTGGGTATGG
**Q-RT-VP39_R**	TTGATGAGGTGGCTGTTGC

Underlined sequence represent Restriction enzyme digestion site.

### Western Blotting

Cells were washed twice with PBS and lysed with 50 μl protein lysis buffer (Beyotime, Shanghai, China) containing 2% PMSF (Beyotime). After 30 min on ice, lysates were treated with 20% loading buffer (Beyotime) for 10 min at 100°C. Proteins were resolved by 15% SDS-PAGE and analyzed by immunoblotting using anti-Flag antibody (1:2000, Sigma, Burghausen, Germany) and anti-GP64 antibody (1:2000, Abcam, Cambridge, USA). The horseradish peroxidase-conjugated goat anti-mouse IgG (1:20000, Beyotime) was used as the secondary antibody. Protein bands were visualized using the Supersignal Western Femto Maximum Sensitivity Substrate Western Blotting Kit (Thermo, Waltham, MA, USA) and a Chemiluminescence Imaging System (Clinx Science Instruments, Shanghai, China).

#### Coimmunoprecipitation (co-IP)

We over-expressed GP64 and BmREEPa simultaneously in BmN-SWU1 cells. At 72 h post-transfection, cells were washed twice with PBS and lysed with 1 ml protein lysis buffer (Beyotime) containing 2% PMSF (Beyotime). After 30 min on ice, lysates were centrifuged at 14000 g for 30 min and the supernatants were collected in a 1.5 ml tube. Then, 50 μl of Sure Beads protein A magnetic beads (Bio-Rad) were transferred to the tube and placed on the magnet to separate the beads from the solution. After removing the supernatant, the tube was taken from the magnet and 5 μg anti-Flag or anti-GP64 antibody diluted in 400 μl PBS with Tween-20 was added to the tube. After gentle pipetting the mixture was incubated with gentle agitation for 30 min at room temperature, and then placed on the magnet to remove the supernatant. The beads were then washed 3 times in PBS. Then, 40 μl of lysis buffer and 10 μl of loading buffer were added and heated for 10 min in a water bath at 100°C. The eluted proteins were detected with anti-GP64 antibody and anti-Flag antibody, respectively.

## Results

### Differential Proteomic Analysis of BmN-SWU1 and BmN-SWU2 Cells

**Fig 1:**
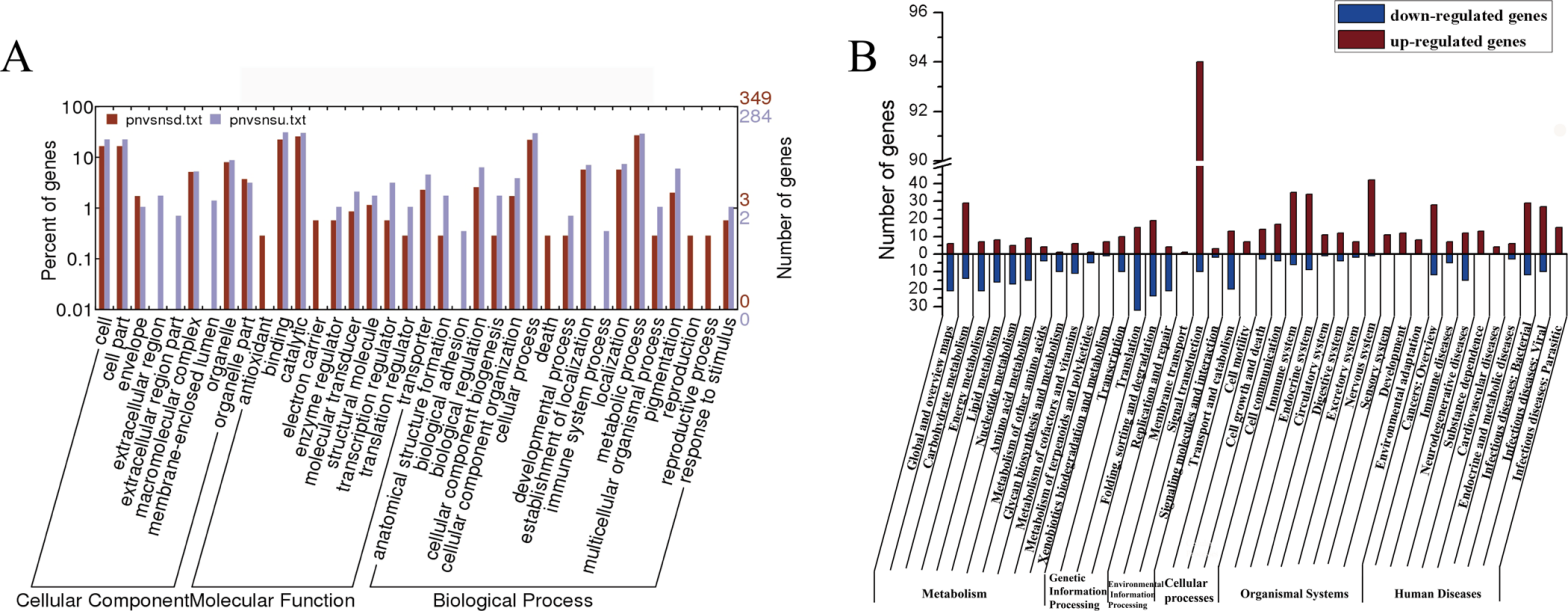
Proteins differently expressed between BmN-SWU1 and BmN-SWU2 via proteomic analysis. (A) GO categories of differently expressed proteins between BmN-SWU1 and BmN-SWU2. A ratio of BmN-SWU2/BmN-SWU1 ≥ 1.5 was considered as up-regulated (brown bar) and ≤ 0.66 was considered down-regulated (purple bar); (B) classification of KEGG pathways associated with the differentially expressed proteins between BmN-SWU1 and BmN-SWU2. The ratio of BmN-SWU2/BmN-SWU1 ≥1.5 was considered up-regulated (brown bar) and ≤ 0.66 was considered down-regulated (blue bar).

The BmN-SWU1 and BmN-SWU2 cell lines were previously established from the silkworm ovary. These two cell lines showed significant differences in their susceptibility to BmNPV infection. To investigate the mechanism by which BmN-SWU2 is refractive to BmNPV, we performed iTRAQ-based differential proteomic analysis using the two cell lines. A total of 629 proteins were differentially expressed between the BmN-SWU2 and BmN-SWU1 cells with 348 up-regulated and 281 down-regulated proteins. GO analysis showed that most of the differentially expressed proteins were involved in the cell, cell part, binding, catalytic, cellular process and metabolic process. Extracellular region category, membrane-enclosed lumen, biological adhesion, and immune system process were unique among the up-regulated proteins while the antioxidant, electron carrier, death, reproduction and reproductive process were specific to the down-regulated proteins (Fig 1A). KEGG analysis showed the involvement of the differentially expressed proteins in most pathways and more than 100 differential proteins were related to signal transduction pathways suggesting that the antiviral mechanisms in BmN-SWU2 cells may involve many cellular processes (Fig 1B).

Previous studies that the process of BmNPV entry into BmN-SWU2 cells occurs in a limited manner [20] suggesting that membrane proteins may be involved in the process. Therefore, we focused our analysis on predicted membrane proteins. Analysis of our proteomic data and previous microarray data identified only four differentially expressed predicted membrane proteins (Table 2). Notably, *BGIBMGA013777* was the only membrane protein with more than a 5-fold difference in expression and therefore the most likely member of the REEP family, which can enhance the receptor function. The result of qRT-PCR showed that the *BGIBMGA013777* expression level in BmN-SWU1 was significantly higher than that in BmN-SWU2 (see S1A Fig). This suggested that this protein could be involved in the entry of BmNPV into BmN-SWU1 and BmN-SWU2 cells.

**Table 2:** Membrane proteins differentially expressed between BmN-SWU1 and BmN-SWU2.

Gene ID	Accession	Gene name
**BGIBMGA013777**	NP_001040329	receptor expression enhancing protein
**BGIBMGA000664**	NP_001040235	uncharacterized protein
**BGIBMGA005451**	NP_001040395	BET1-like protein
**BGIBMGA010376**	NP_001108406	uncharacterized protein

### Cloning of *BmREEPa*

**Fig 2:**
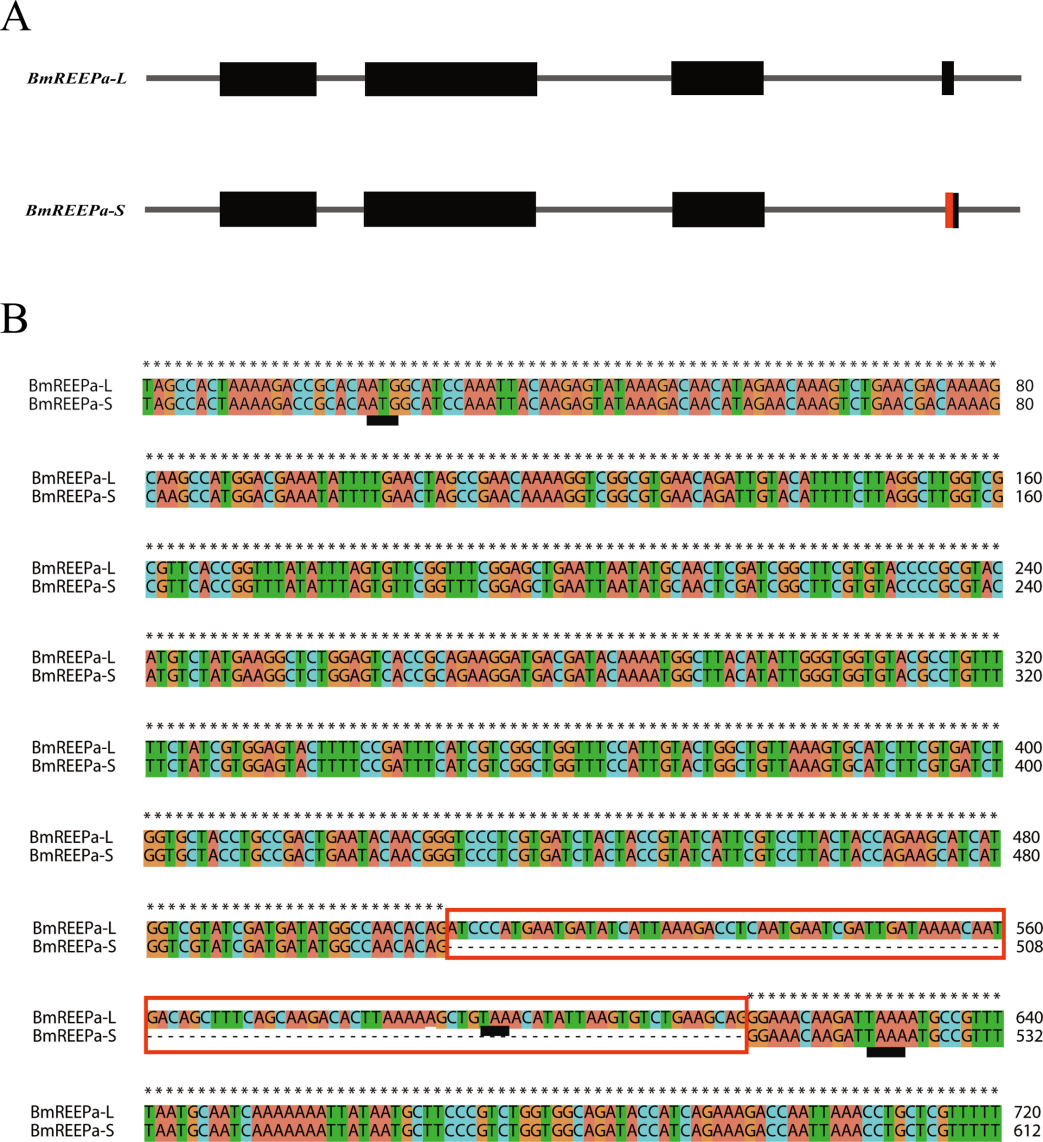
Two different splicesomes of *BmREEPa*. (A) Gene structure of two *BmREEPa* splicesomes. The red region is lacking in *BmREEPa*-S. (B) Differences between the gene sequences of the two *BmREEPa* splicesomes; the sequence in red is not present in *BmREEPa*-S; black underline represents the start and stop codons.

**Fig 3:**
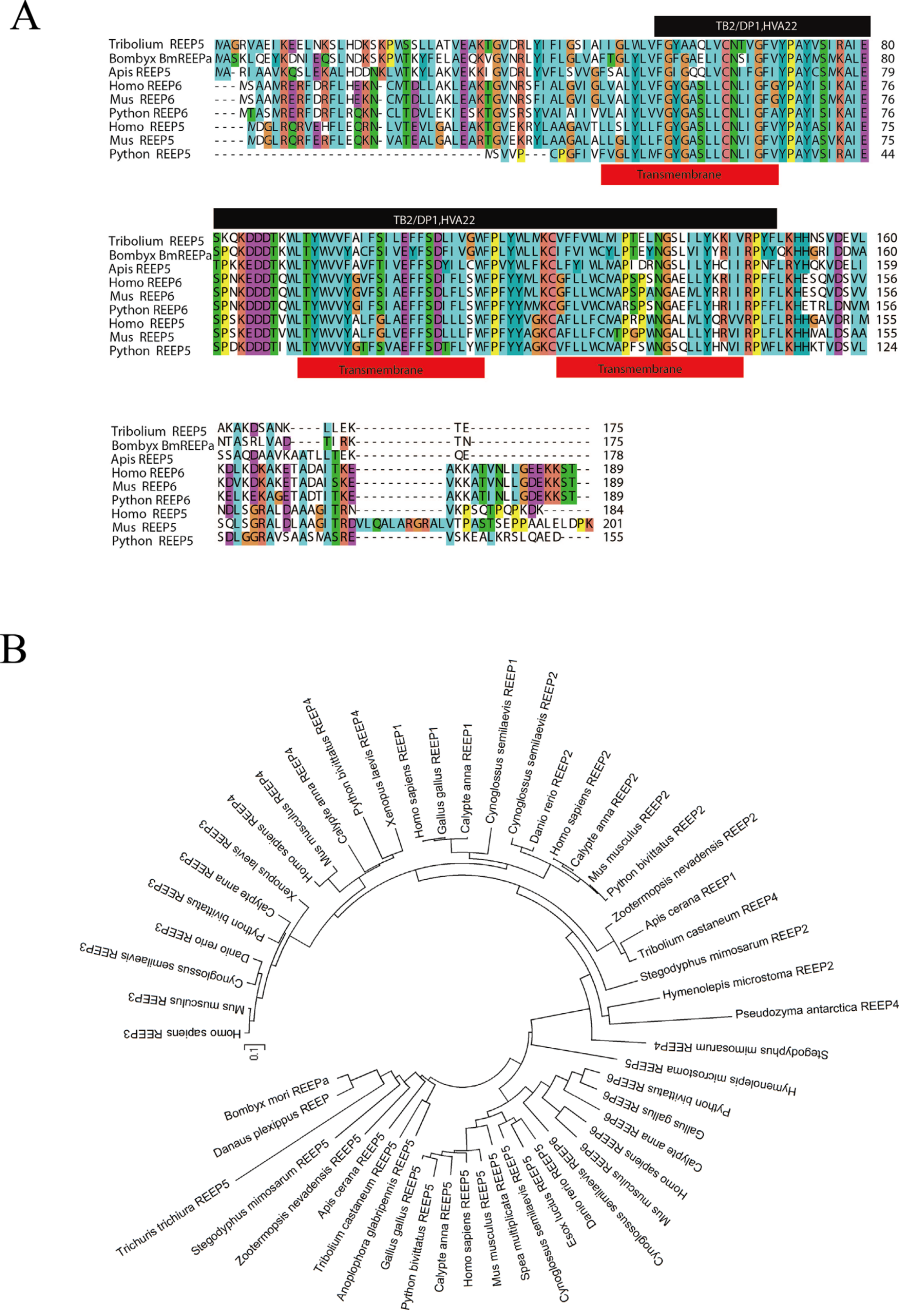
Homology analysis of BmREEPa. (A) Sequence analysis of BmREEPa. Black color represents the TB2/DP1, HVA22 domain; red represents transmembrane domain; (B) phylogenetic tree (N-J) of BmREEPa with REEP genes from vertebrates and invertebrates.

*BGIBMGA013777* was cloned from P50 silkworm larvae. We obtained two splice variants for *BmREEPa*; *BmREEPa-L* and *BmREEPa-S* (GenBank accession number: KR260842, KR260843). The coding sequence (CDS) of *BmREEPa-L* is 573 bp long and encodes a putative protein with 190 amino acids while the CDS of *BmREEPa-S* is 501 bp long and encodes 166 amino acids. We observed that a 108 bp deletion in the fourth exon of *BmREEPa-S* shifted the stop codon thus resulting in a longer BmREEPa-L protein (Fig 2A and 2B). In addition, the *BmREEPa* homolog in the BmN-SWU1 cells and silkworm larvae had mutations only in 8 bp thus generating 3 differences in the predicted amino acid sequence. Our attempt to clone *BmREEPa* from BmN-SWU2 cells was unsuccessful likely because of low *BmREEPa* expression levels in the cells. This gene showed 93% similarity to the *Danaus plexippus* REEP protein and was 58% and 61% similar to human REEP5 and REEP6, respectively. Sequence analysis showed that BmREEPa had a TB2/DP1, HVA22 domain, which is conserved in the REEP protein family as well as three transmembrane domains (Fig 3A). Based on these findings, we named the resulting sequence as *BmREEPa*. Phylogenetic analysis showed that the deduced amino acid sequence of BmREEPa clustered with the REEP5-REEP6 subfamily, and that each subfamily was divided into vertebrate and invertebrate subgroups (Fig 3B). BmREEPa showed the highest homology with *D*. *plexippus* REEP. These results also suggest that BmREEPa is homologous to the human REEP5 and REEP6 proteins.

### BmREEPa Is Localized in the Cell Membrane

**Fig 4:**
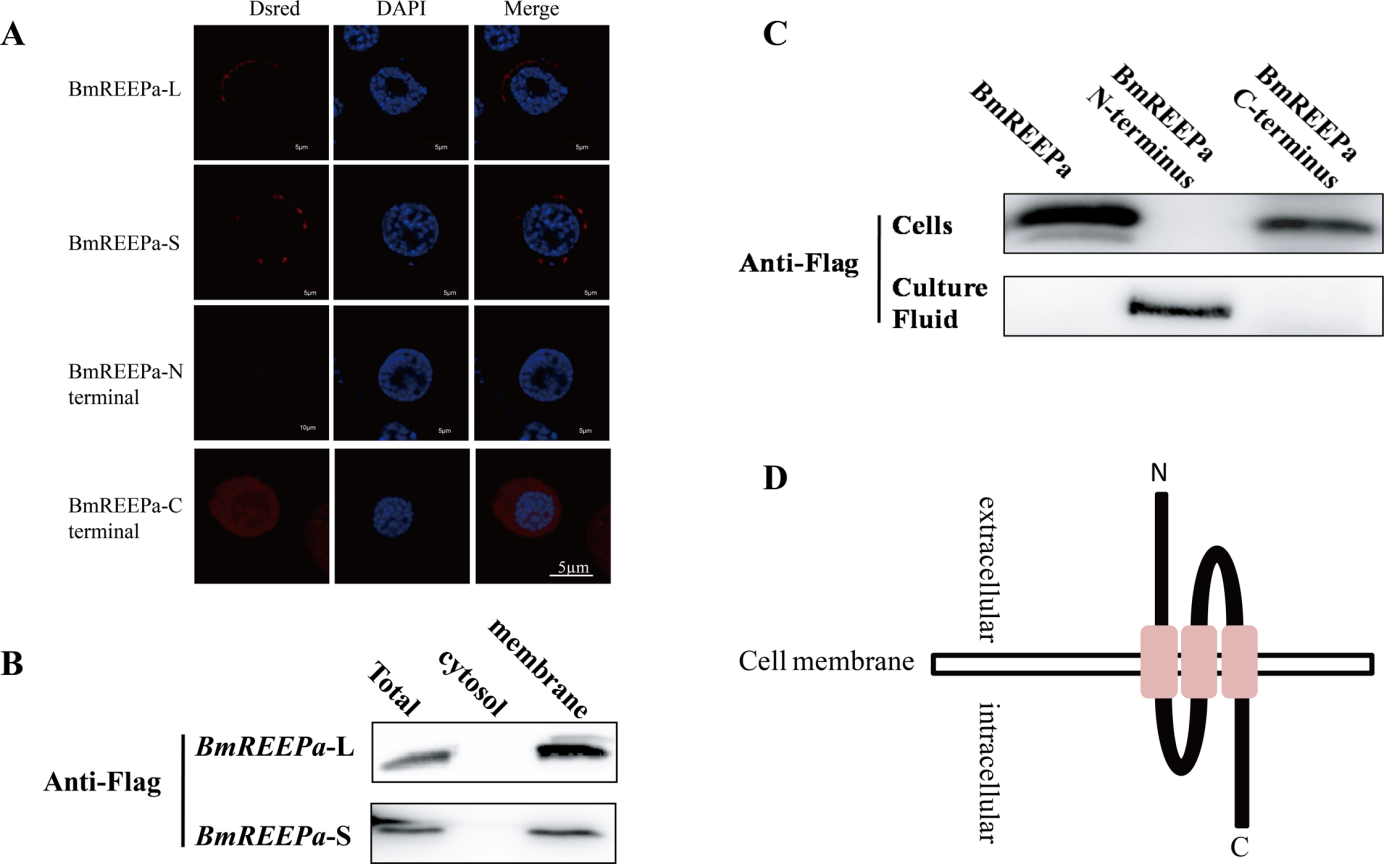
BmREEPa localization and topology model. (A) BmREEPa full-length, N-terminal and C-terminal sequence localization; (B) Western blot of full-length BmREEPa in the isolated membrane and cytosol proteins; (C) Western blot of BmN-SWU1 cells and culture fluid expressing BmREEPa N- and C-termini; (D) Topology model of BmREEPa. BmREEPa-L, BmREEPa-S, BmREEPa-N terminus and BmREEPa-C terminus were co-expressed with Flag tags and DsRed. DsRed was used as a marker in fluorescence observation while anti-Flag antibody was used in Western blots.

BmN-SWU1 cells were transfected with a vector co-expressing BmREEPa and DsRed, and then were examined through a laser scanning confocal microscope. The results showed localization of BmREEPa in the cell membrane with punctate distribution (Fig 4A). To further validate these results, total proteins were extracted from the cells and separated into membrane proteins and cytosol proteins using Membrane and Cytosol Protein Extraction Kit (Beyotime) for analysis by Western Blotting. BmREEPa was detected only in the membrane protein and total protein consistent with microscopic observations (Fig 4B). To explore the topology of BmREEPa in the cell membrane, the C- (1−47aa) and N-termini (145−190aa) of BmREEPa were separately fused to DsRed and expressed in BmN-SWU1 cells (S2 Fig). Red fluorescence was observed only in cells expressing the BmREEPa C-terminus fused to DsRed, but not in cells expressing the N-terminus DsRed fusion protein (Fig 4A) indicating that the N-terminus of the protein is extracellular and the C-terminus is cytoplasmic. Western blot analysis showed that the C-terminus DsRed fusion protein and full-length DsRed fusion protein were detected in the transfected cells and the N-terminus DsRed fusion protein was detected in the cell culture fluid consistent with fluorescence microscopic observations (Fig 4C). These results indicate that BmREEPa is localized in the cell membrane with its C-terminus in the cytoplasm and the N-terminus outside the cell (Fig 4D).

### BmREEPa Affects the Infection of BmNPV in BmN-SWU1 and BmN-SWU2 Cells

**Fig 5:**
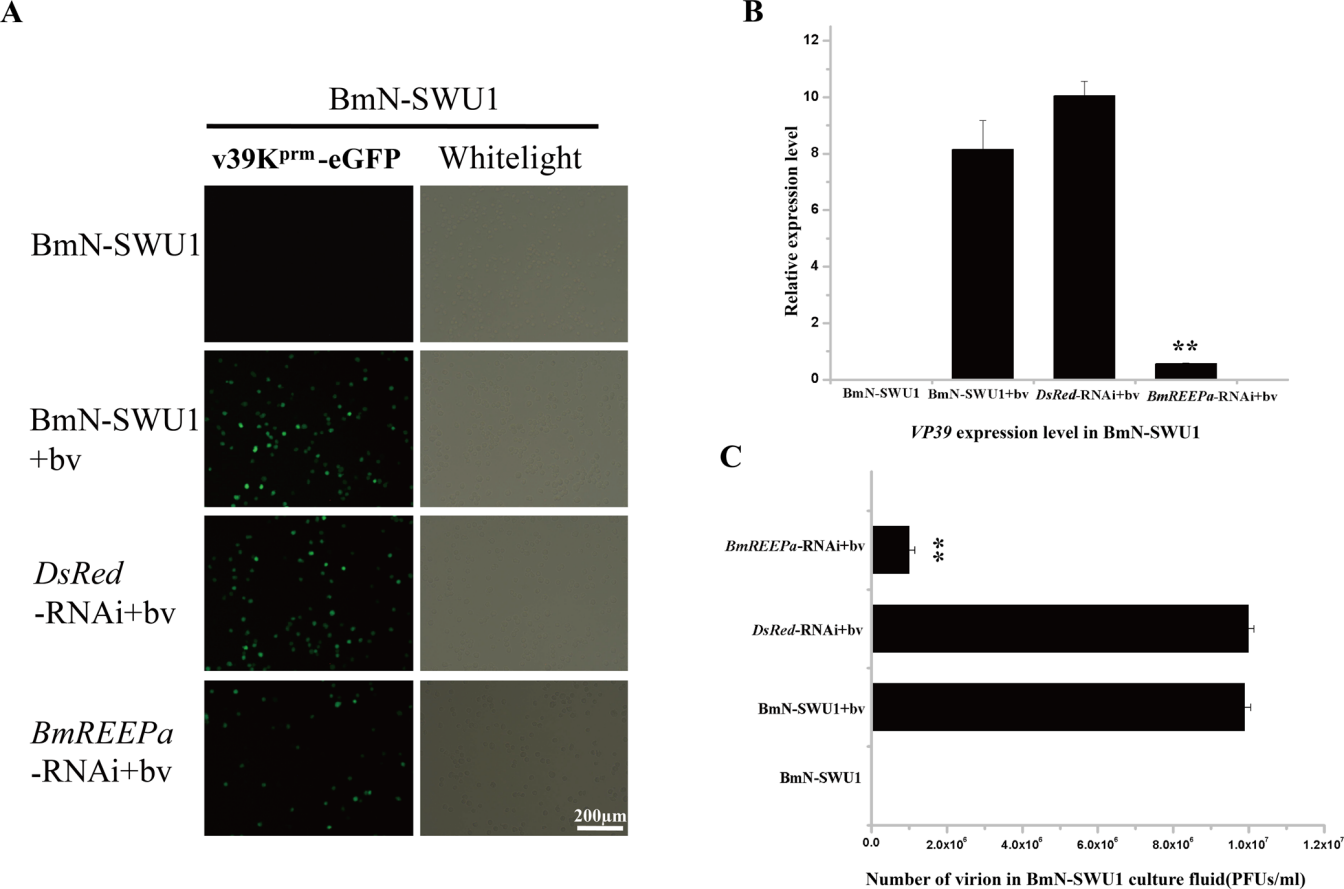
Effect of BmNPV transduction in *BmREEPa*-RNAi BmN-SWU1 cells. (A) Green fluorescence in cells at 48 h post-infection with v39K^prm^-eGFP BVs; (B) Analysis of *VP39* expression in BmN-SWU1 48 h after infection with v39K^prm^-eGFP BVs; (C) Viral titer in cell culture fluid 48 h after infection with v39K^prm^-eGFP BVs. BmN-SWU1 without v39K^prm^-eGFP BVs, BmN-SWU1 with v39K^prm^-eGFP BVs and DsRed-RNAi with v39K^prm^-eGFP BVs were used as controls; * indicates significant differences at P < 0.05, ** indicates significant differences at P < 0.01 with respect to the control.

**Fig 6:**
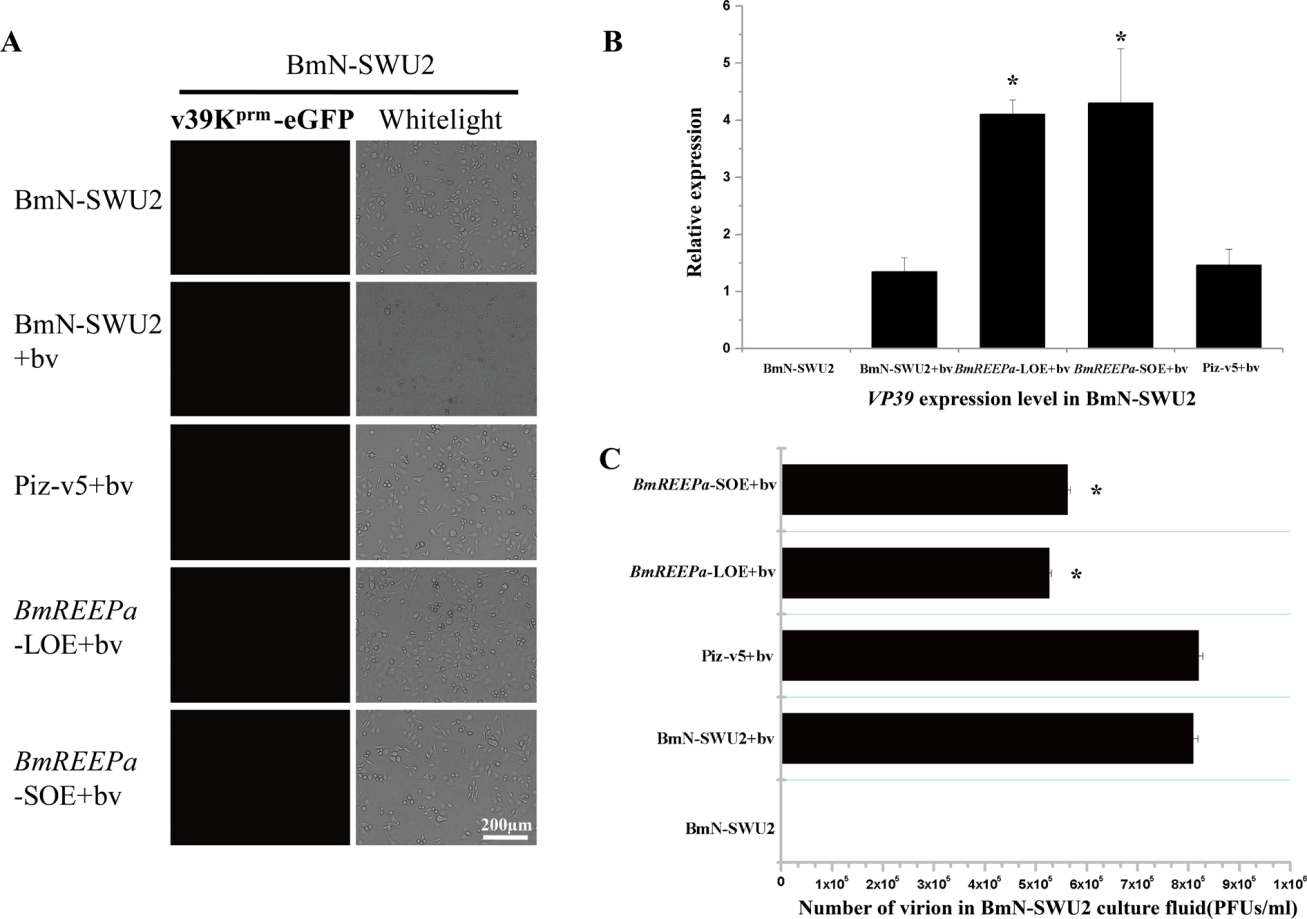
Effect of BmNPV transduction in BmN-SWU2 cells over-expressing *BmREEPa*. (A) Green fluorescence in cells infected with v39K^prm^-eGFP BVs; (B) Analysis of *VP39* expression in cells 48 h after infection with v39K^prm^-eGFP BVs; (C) Viral titer in cell culture fluid at 48 h post-infection with v39K^prm^-eGFP BVs. BmN-SWU2 without v39K^prm^-eGFP BVs, BmN-SWU2 with v39K^prm^-eGFP BVs and with v39K^prm^-eGFP BVs were used as controls; * indicates significant differences at P < 0.05, ** indicates significant differences at P < 0.01 when compared to the control.

Our study showed that BmREEPa expression level was significantly higher in BmN-SWU1 than BmN-SWU2 cells. Therefore, we inhibited *BmREEPa* in BmN-SWU1 cells by transfecting with pIZ-V5-BmREEPa-RNAi vector (see S1B Fig) following by the addition of v39K^prm^-eGFP BVs after 48 h. Fluorescence was observed 48 h post-infection. Then, we collected the cells and culture fluid separately to examine *VP39* expression and viral titer. VP39 is a late baculovirus capsid gene widely used as a marker of BmNPV. The results showed that RNAi of *BmREEPa* in BmN-SWU1 significantly decreased the number of green fluorescence positive virus-infected cells when compared to the control BmN-SWU1 cells (Fig 5A). The expression of *VP39* was markedly decreased in *BmREEPa*-inhibited BmN-SWU1 compared to the control BmN-SWU1 cells (Fig 5B). The viral titer of BmNPV in the culture fluid of *BmREEPa-*inhibited BmN-SWU1 cells was also significantly lower than in the control BmN-SWU1 culture fluid (Fig 5C). These results indicate that *BmREEPa* depletion by RNAi can inhibit the infection of BmNPV in these cells. Furthermore, we over-expressed *BmREEPa* in BmN-SWU2 cells by transfecting the cells with pIZ-V5-BmREEPa-L/-S vector (see S1C Fig) following by infection with v39K^prm^-eGFP BVs after 48 h. After detecting fluorescence 48 h post-infection, the cells and culture fluid were collected individually to examine the *VP39* expression level and viral titer. Green fluorescence was not detected in the control BmN-SWU2 as well as the *BmREEPa* over-expressing BmN-SWU2 cells (Fig 6A). However, *VP39* expression was higher in *BmREEPa* over-expressing BmN-SWU2 cells than in control BmN-SWU2 cells. Moreover, over-expression of both *BmREEPa*-L and *BmREEPa*-S had similar effects (Fig 6B). In addition, BmNPV titer in the culture fluid of BmN-SWU2 cells over-expressing BmREEPa was lower than in the control BmN-SWU2 cells (Fig 6C) suggesting that the ability of virus to enter BmN-SWU2 cells over-expressing *BmREEPa* was higher than the control BmN-SWU2 cells. These results indicate that although BmNPV can enter BmN-SWU2 cells, the virus may be unable to complete its replication in these cells. In short, BmREEPa can affect BmNPV infection in BmN-SWU1 and BmN-SWU2 cells.

### BmREEPa Influences BmNPV Entry into BmN-SWU1 and BmN-SWU2 Cells

**Fig 7:**
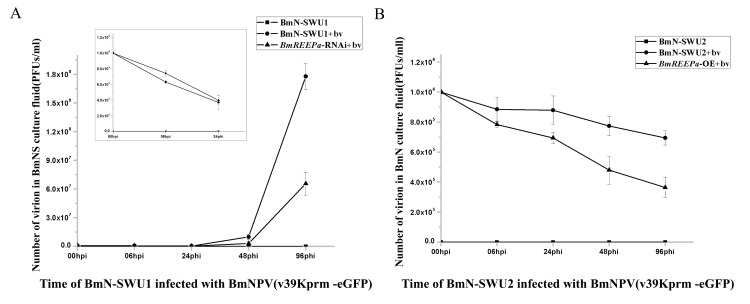
Viral titer at different stages of infection. (A) Viral titer in *BmREEPa*-RNAi BmN-SWU1 and control BmN-SWU1 cell culture fluid at 0 h, 6 h, 24 h, 48 h and 96 h after infection with v39K^prm^-eGFP BVs; (B) Viral titer in *BmREEPa* over-expressing BmN-SWU2 and control BmN-SWU2 cell culture fluid at 0 h, 6 h, 24 h, 48 h and 96 h after infection with v39K^prm^-eGFP BVs. BmN-SWU1 and BmN-SWU2 without v39K^prm^-eGFP BVs, BmN-SWU1 and BmN-SWU2 with v39K^prm^-eGFP BVs were used as controls.

**Fig 8:**
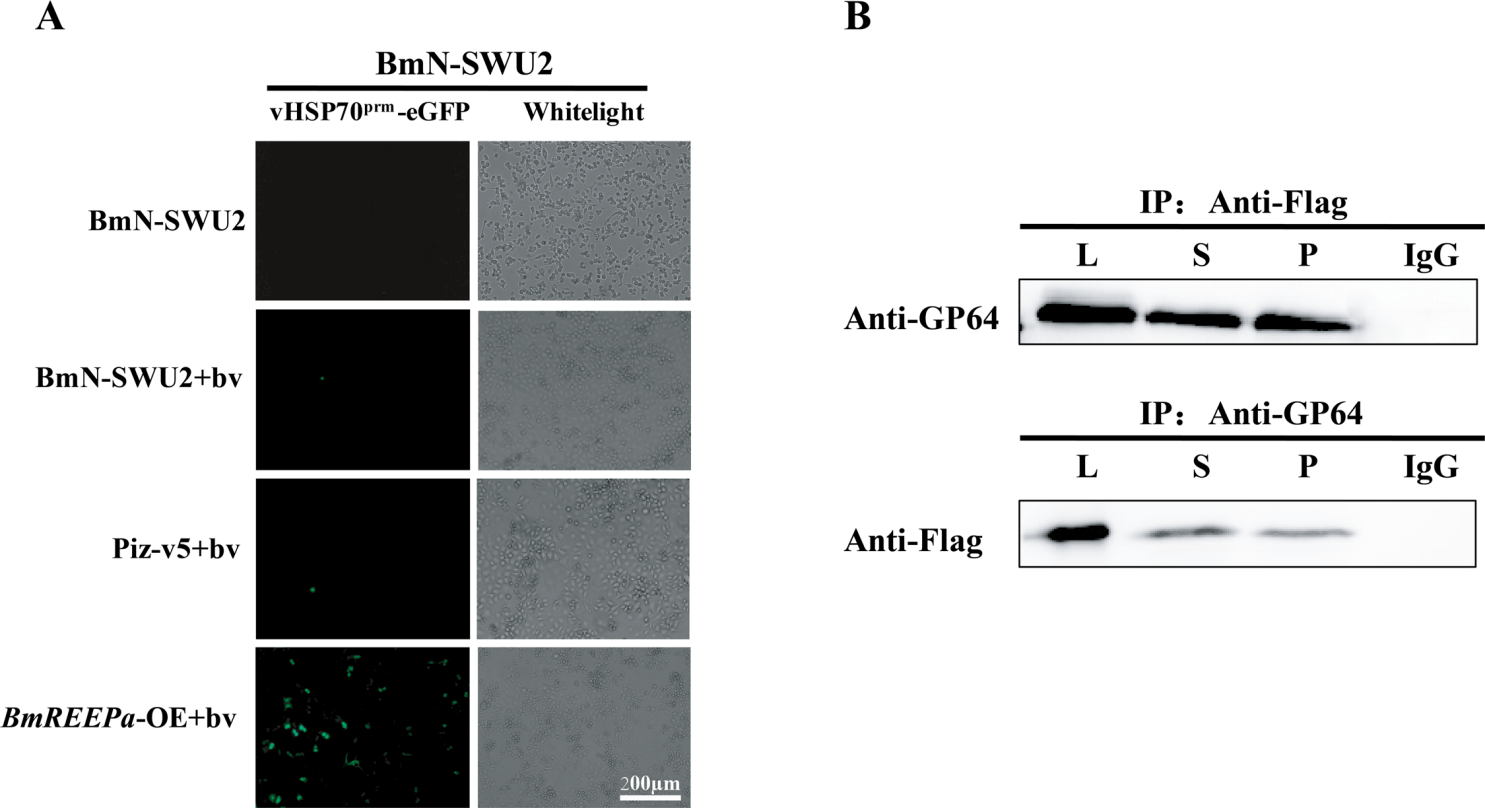
Function of *BmREEPa* during BmNPV entry. **(A)** Green fluorescence in BmN-SWU2 infected with vHSP70^prm^-eGFP BVs, BmN-SWU2 without vHSP70^prm^–eGFP BVs, BmN-SWU2 with vHSP70^prm^-eGFP BVs and pIZ-V5/his with vHSP70^prm^-eGFP BVs were used as controls; (B) Western blot analysis determining the interaction between BmREEPa and GP64; P indicates lysate, S supernatant, P pellet; IgG was used as the control.

Previous studies showed that the entry of BmNPV into BmN-SWU2 was suppressed [20]. Our data shows that the membrane protein, BmREEPa, can affect BmNPV infection, suggesting that BmREEPa may play a role in the entry of BmNPV into cells. To test this hypothesis, we compared the BmNPV titer in the cell culture fluid of *BmREEPa*-inhibited BmN-SWU1, *BmREEPa* over-expressing BmN-SWU2 and control BmN-SWU2 after 0, 6, 24, 48 and 96 h of viral infection, respectively. The results showed that 0−24 h after BmNPV infection the viral titer in the culture fluid with the anti-sense *BmREEPa* RNA was higher than in the control cells without RNAi suggesting that more viruses entered the control BmN-SWU1 cells than the BmN-SWU1 cells with anti-sense *BmREEPa* RNA. However, 24−96 h after infection, the viral titer in the RNAi group was lower than the control cells likely due to more viral production in the control BmN-SWU1 cells than in *BmREEPa* depleted BmN-SWU1 cells (Fig 7A). These results suggest that *BmREEPa* RNAi can suppress BmNPV entry into BmN-SWU1 cells. In addition, 0−96 h after viral infection, the culture fluid in *BmREEPa* over-expressing BmN-SWU2 cells consistently showed lower viral titers than the control BmN-SWU2 cells(Fig 7B) corroborating the above results that *BmREEPa* may play a positive role in viral entry into BmN-SWU2 cells. However, we did not detect fluorescence in the cells over-expressing *BmREEPa* probably because the 39K promoter was not induced by the virus. In order to explain this, we infected the BmN-SWU2 and *BmREEPa* over-expressing BmN-SWU2 cells with vHSP70^prm^-eGFP BVs, which express eGFP immediately after the entry of BmNPV into cells. More green fluorescence was detected in *BmREEPa* over-expressing BmN-SWU2 cells and only limited green fluorescence was detected in control BmN-SWU2 (Fig 8A). This result suggested that *BmREEPa* can rescue the limitation of BmNPV entry into BmN-SWU2.

### BmREEPa Can Interact with GP64

The glycoprotein GP64, a major envelope fusion protein, is essential to internalize a virus into a host cell [24–29]. In this study we found that BmREEPa can affect BmNPV entry into silkworm cells. To examine whether it can interact with GP64, we simultaneous over-expressed GP64 and BmREEPa with Flag tags in BmN-SWU1 cells, then examined their relationship by co-IP. The results showed that BmREEPa was detected in the group where GP64 was used as a decoy protein; similarly, GP64 was detected when BmREEPa was used as a decoy protein (Fig 8B). These findings indicated that BmREEPa can interact with the BmNPV envelope protein, GP64, thus providing further evidence for the role of BmREEPa in the entry of BmNPV into silkworm cells.

## Discussion

Two silkworm cell lines, BmN-SWU1 and BmN-SWU2 [19], were previously shown to have differential susceptibility to BmNPV infection [20]. In order to understand the mechanism underlying these differential properties, we compared the proteomes of BmN-SWU1 and BmN-SWU2 cells by iTRAQ-based proteomic technology. Among the differentially expressed proteins we identified a membrane protein named BmREEPa. We characterized the *BmREEPa* gene and identified the following properties in its putative protein; sequence analysis revealed that BmREEPa contains a highly conserved domain characteristic of the REEP family (TB2/DP1, HVA22) and three transmembrane domains; phylogenetic analysis demonstrated that BmREEPa belongs to the REEP5-REEP6 subfamily; BmREEPa was localized on the cell membrane and exhibited a unique topology with the N-terminus being extracellular and the C-terminus being cytoplasmic. Thus, the BmREEPa gene has the characteristics of the REEP family particularly the REEP5-REEP6 subfamily.

REEPs are a group of proteins, which enhance the expression of cell surface receptors and are involved in the transport of transmembrane proteins. They belong to the DP1/Yop1p family and maintain a high degree of homology between different species (Fig 3A) [9]. Previous studies largely focused on the REEP1-REEP4 subfamily with most exploring the role of REEPs in human diseases. However, to our knowledge no study has shown the involvement of REEPs in viral infection. In the present study, we found that after BmNPV infection, *BmREEPa*-depleted BmN-SWU1 cells had significantly lesser number of virus-infected cells and viral particles, and lower *VP39* expression level in comparison to the control BmN-SWU1 cells. These results suggest that *BmREEPa* RNAi inhibited the infection of BmNPV in BmN-SWU1 cells. In the virus resistant BmN-SWU2 cells, no green fluorescence positive BmN-SWU2 cells were detected regardless of whether *BmREEPa* was over-expressed or inhibited. However, BmN-SWU2 cells over-expressing *BmREEPa* had higher *VP39* expression and lesser viral particles in the culture fluid than the control BmN-SWU2 cells. Furthermore, we used vHSP70^prm^-eGFP BVs, which expressed eGFP without BmNPV induction to infect BmREEPa over-expressing BmN-SWU2 and found that more green fluorescence can be detected while the control BmN-SWU2 had only a few green fluorescent cells. Based on these results we presume that in *BmREEPa* over-expressing BmN-SWU2 cells BmNPV can gain entry into cells easily, but cannot complete the viral replication process. Time course analysis of viral titer in the culture fluid revealed that *BmREEPa* RNAi markedly decreased viral entry into BmN-SWU1 cells, while *BmREEPa* over-expression increased virus entry into BmN-SWU2 cells. Unlike BmN-SWU1 cells, the number of viral particles did not increase after 24 h post-infection but kept declining in BmN-SWU2 cells over-expressing *BmREEPa* suggesting that BmNPV can enter into BmN-SWU2 cells but cannot proliferate in them. Previous studies showed that REEPs enhanced receptor function by recruiting receptors to lipid rafts, rather than directly increasing the expression of receptors [9,17]. We presume that BmREEPa may also enhance potential BmNPV receptors in a similar manner. We also found that BmREEPa can interact with GP64, which is an essential gene for BmNPV entry. In this study, we hypothesized that BmNPV can enter BmN-SWU2 cells over-expressing *BmREEPa* but cannot complete their replication in them. Our results indicate that BmNPV transportation or transcription/translation processes are likely inhibited in these cells. Taken together, we suspected that BmREEPa can recruit BmNPV receptor, which may be a protein or small molecule, to the rafts in the plasma membrane to form a protein-protein or protein-small molecule complex. Then, GP64 interacts with the complex and helps virions to enter cells via receptor-mediated endocytosis during the process of BmNPV entry.

In conclusion, we identified a key gene named *BmREEPa*, which regulates the entry of BmNPV into silkworm cells. BmREEPa is localized in the cell membrane, with the C-terminus in the cytoplasm and the N-terminus outside the cell. This is the first study to report the role of an REEP gene in viral infection and our study provides a new insight for the identification of BmNPV receptor and the exploration of BmNPV infection mechanism.

## Supporting Information

S1 Fig Expression level of BmREEPa. (A) Different BmREEPa expression level in BmN-SWU1 and BmN-SWU2; (B) BmREEPa expression level in BmN-SWU1 and BmREEPa inhibited BmN-SWU1; (C) BmREEPa expression level in BmN-SWU2 and BmREEPa over-expressed BmN-SWU2.
(TIF)

S2 Fig Schematic diagram of BmREEPa. (TIF)

## References

[1] Lin Y, Liu Z, Liu X, Zhang Y, Rong Z, Li D. (2013) Microarray-based analysis of the gene expression profile in GC-1 spg cells transfected with spermatogenesis associated gene 12. Int J Mol Med 31: 459–466. 10.3892/ijmm.2012.1225 23292202

[2] Yao HP, Chen L, Xiang X, Guo AQ, Lu XM, Wu XF. (2010) Proteomics identification and annotation of proteins of a cell line of Bombyx mori, BmN cells. Biosci Rep 30: 209–215. 10.1042/BSR20090045 19496755

[3] Zhou Y, Gao L, Shi H, Xia H, Gao L, Lian C, et al (2013) Microarray analysis of gene expression profile in resistant and susceptible Bombyx mori strains reveals resistance-related genes to nucleopolyhedrovirus. Genomics 101: 256–262. 10.1016/j.ygeno.2013.02.004 23434630

[4] Sagisaka A, Fujita K, Nakamura Y, Ishibashi J, Noda H, Imanishi S, et al (2010) Genome-wide analysis of host gene expression in the silkworm cells infected with Bombyx mori nucleopolyhedrovirus. Virus Res 147: 166–175. 10.1016/j.virusres.2009.10.015 19883703

[5] Qin L, Xia H, Shi H, Zhou Y, Chen L, Yao Q, et al (2012) Comparative proteomic analysis reveals that caspase-1 and serine protease may be involved in silkworm resistance to Bombyx mori nuclear polyhedrosis virus. J Proteomics 75: 3630–3638. 10.1016/j.jprot.2012.04.015 22546490

[6] Salem TZ, Zhang F, Xie Y, Thiem SM (2011) Comprehensive analysis of host gene expression in Autographa californica nucleopolyhedrovirus-infected Spodoptera frugiperda cells. Virology 412: 167–178. 10.1016/j.virol.2011.01.006 21276998 PMC3056905

[7] Selot R, Kumar V, Sekhar SC, Kumar PG (2010) Molecular characterization and expression analysis of BmNOX in two strains of Bombyx mori with contrasting viral resistance phenotype. Arch Insect Biochem Physiol 73: 163–175. 10.1002/arch.20348 20077572

[8] Tavares J, Bravo JP, Gimenes F, Neto QA, Fiorini A, Fernadez MA,. (2011) Differential structure of the intronic promoter of the Bombyx mori A3 actin gene correlated with silkworm sensitivity/resistance to nucleopolyhedrovirus. Genet Mol Res 10: 471–481. 10.4238/vol10-1gmr978 21476193

[9] Bjork S, Hurt CM, Ho VK, Angelotti T (2013) REEPs are membrane shaping adapter proteins that modulate specific g protein-coupled receptor trafficking by affecting ER cargo capacity. PLoS One 8: e76366 10.1371/journal.pone.0076366 24098485 PMC3788743

[10] Hurt CM, Bjork S, Ho VK, Gilsbach R, Hein L, Angelotti T. (2014) REEP1 and REEP2 proteins are preferentially expressed in neuronal and neuronal-like exocytotic tissues. Brain Res 1545: 12–22. 10.1016/j.brainres.2013.12.008 24355597 PMC3919455

[11] Argasinska J, Rana AA, Gilchrist MJ, Lachani K, Young A, Smith JC. (2009) Loss of REEP4 causes paralysis of the Xenopus embryo. Int J Dev Biol 53: 37–43. 10.1387/ijdb.072542ja 19123125

[12] Esteves T, Durr A, Mundwiller E, Loureiro JL, Boutry M, Gonzalez MA, et al (2014) Loss of association of REEP2 with membranes leads to hereditary spastic paraplegia. Am J Hum Genet 94: 268–277. 10.1016/j.ajhg.2013.12.005 24388663 PMC3928657

[13] Mainland J, Matsunami H (2012). RAMP like proteins, RTP and REEP family of proteins, in Spielman W. S., Parameswaran N., (Eds.), Advances in Experimental Medicine and Biology, RAMPs, Vol. 744 Springer Science+Business Media, New York, pp. 75–86. 10.1007/978-1-4614-2364-5_7 22434109

[14] Beetz C, Schule R, Deconinck T, Tran-Viet KN, Zhu H, Kremer BP, et al (2008) REEP1 mutation spectrum and genotype/phenotype correlation in hereditary spastic paraplegia type 31. Brain 131: 1078–1086. 10.1093/brain/awn026 18321925 PMC2841798

[15] Castermans D, Vermeesch JR, Fryns JP, Steyaert JG, Van de Ven WJ, Greemers JW, et al (2007) Identification and characterization of the TRIP8 and REEP3 genes on chromosome 10q21.3 as novel candidate genes for autism. Eur J Hum Genet 15: 422–431. 17290275 10.1038/sj.ejhg.5201785

[16] Du J, Shen L, Wang YG, Liao SS, Chen C, Zhou ZF, et al (2009) Receptor expression-enhancing protein 1 gene (SPG31) mutations are rare in Chinese Han patients with hereditary spastic paraplegia. Chinese Medical Journal 122. 19781397

[17] Ilegems E, Iwatsuki K, Kokrashvili Z, Benard O, Ninomiya Y, Margolskee RF. (2010) REEP2 enhances sweet receptor function by recruitment to lipid rafts. J Neurosci 30: 13774–13783. 10.1523/JNEUROSCI.0091-10.2010 20943918 PMC3168766

[18] Yang Z, Ma X, Wang Y, Wang J, Xiang B, Wu J, et al (2012) Association of APC and REEP5 gene polymorphisms with major depression disorder and treatment response to antidepressants in a Han Chinese population. Gen Hosp Psychiatry 34: 571–577. 10.1016/j.genhosppsych.2012.05.015 22795047

[19] Pan MH, Cai XJ, Liu M, Lv J, Tang H, Tan J, et al (2010) Establishment and characterization of an ovarian cell line of the silkworm, Bombyx mori. Tissue Cell 42: 42–46. 10.1016/j.tice.2009.07.002 19665160

[20] Zhang J, Chen XM, Zhang CD, He Q, Dong ZQ, Cao MY, et al (2014) Differential susceptibilities to BmNPV infection of two cell lines derived from the same silkworm ovarian tissues. PLoS One 9: e105986 10.1371/journal.pone.0105986 25221982 PMC4164443

[21] Zhang J, He Q, Zhang CD, Chen XY, Chen XM, Pan MH, et al (2014) Inhibition of BmNPV replication in silkworm cells using inducible and regulated artificial microRNA precursors targeting the essential viral gene lef-11. Antiviral Res 104: 143–152. 10.1016/j.antiviral.2014.01.017 24486953

[22] Shen H, Chen K. (2012 **)** BM61 of Bombyx mori nucleopolyhedrovirus: its involvement in the egress of nucleocapsids from the nucleus. FEBS letters, 586, 990–995. 10.1016/j.febslet.2011.12.040 22569252

[23] Wang W, Huang MH, Dong XL, Chai CL, Pan CX, Tang H, et al (2014) Combined Effect of Cameo2 and CBP on the Cellular Uptake of Lutein in the Silkworm,Bombyx mori. PLoS One 9. 10.1371/journal.pone.0086594 PMC3903547 24475153

[24] De Jong J, Theilmann DA, Arif BM, Krell PJ (2011) Immediate-early protein ME53 forms foci and colocalizes with GP64 and the major capsid protein VP39 at the cell membranes of Autographa californica multiple nucleopolyhedrovirus-infected cells. J Virol 85: 9696–9707. 10.1128/JVI.00833-11 21775466 PMC3196415

[25] Kataoka C, Kaname Y, Taguwa S, Abe T, Fukuhara T, Tani H, et al (2012) Baculovirus GP64-mediated entry into mammalian cells. J Virol 86: 2610–2620. 10.1128/JVI.06704-11 22190715 PMC3302255

[26] Li Z, Blissard GW (2010) Baculovirus GP64 disulfide bonds: the intermolecular disulfide bond of Autographa californica multicapsid nucleopolyhedrovirus GP64 is not essential for membrane fusion and virion budding. J Virol 84: 8584–8595. 10.1128/JVI.00264-10 20573818 PMC2918989

[27] Li Z, Blissard GW (2011) Autographa californica multiple nucleopolyhedrovirus GP64 protein: roles of histidine residues in triggering membrane fusion and fusion pore expansion. J Virol 85: 12492–12504. 10.1128/JVI.05153-11 21937651 PMC3209339

[28] Dong S, Blissard GW (2012) Functional analysis of the Autographa californica multiple nucleopolyhedrovirus GP64 terminal fusion loops and interactions with membranes. J Virol 86: 9617–9628. 10.1128/JVI.00813-12 22740400 PMC3446601

[29] Luz-Madrigal A, Asanov A, Camacho-Zarco AR, Sampieri A, Vaca L (2013) A cholesterol recognition amino acid consensus domain in GP64 fusion protein facilitates anchoring of baculovirus to mammalian cells. J Virol 87: 11894–11907. 10.1128/JVI.01356-13 23986592 PMC3807332

