# Supplementary figures and images for: BmREEPa Is a Novel Gene that Facilitates BmNPV Entry into Silkworm Cells
Source: PLoS One. 2015 Dec 14;10(12):e0144575. doi: 10.1371/journal.pone.0144575 (PMC4681539; doi:10.1371/journal.pone.0144575)

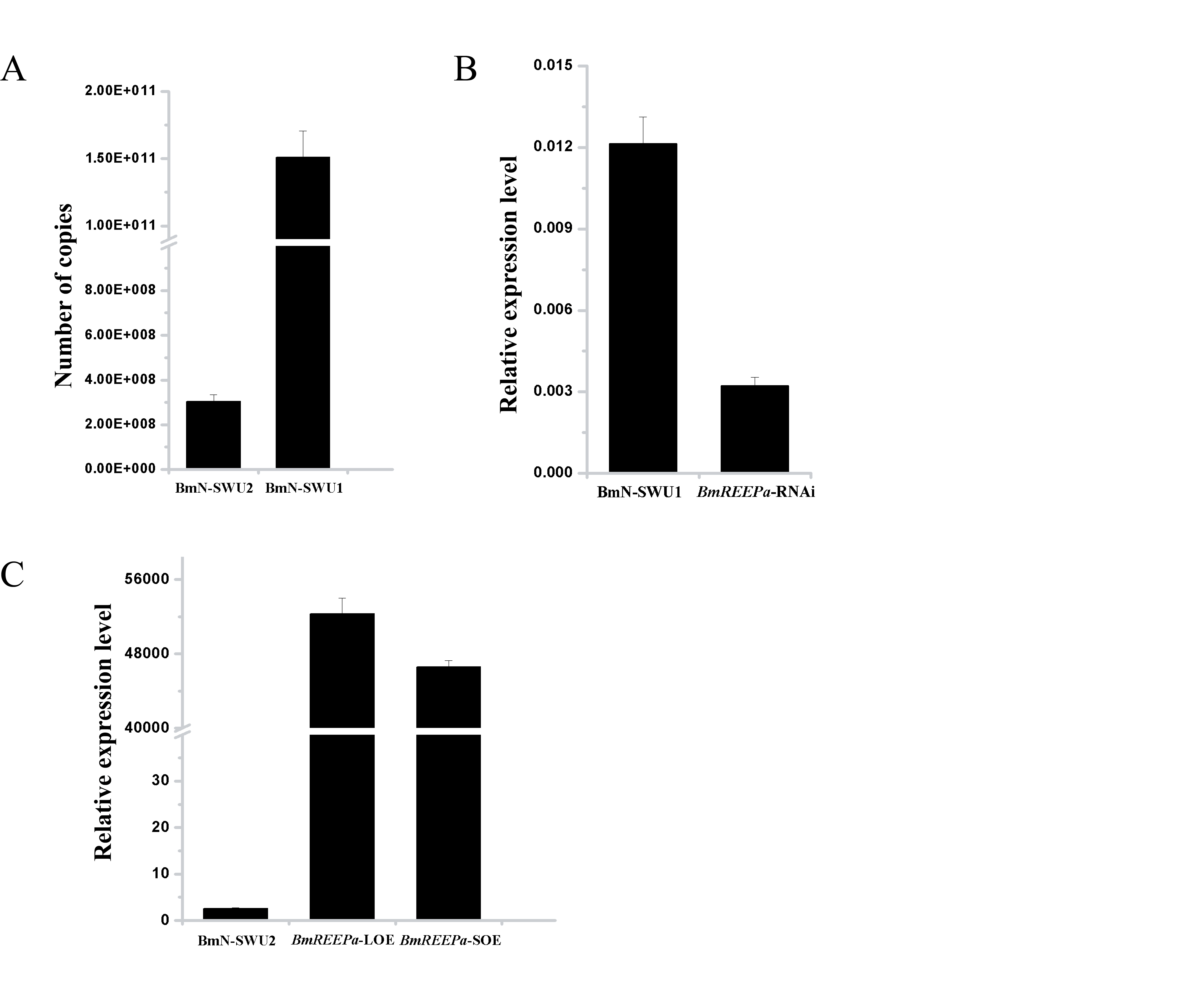

Supplement: S1 Fig — (A) Different BmREEPa expression level in BmN-SWU1 and BmN-SWU2; (B) BmREEPa expression level in BmN-SWU1 and BmREEPa inhibited BmN-SWU1; (C) BmREEPa expression level in BmN-SWU2 and BmREEPa over-expressed BmN-SWU2. (TIF) [file pone.0144575.s001.tif]

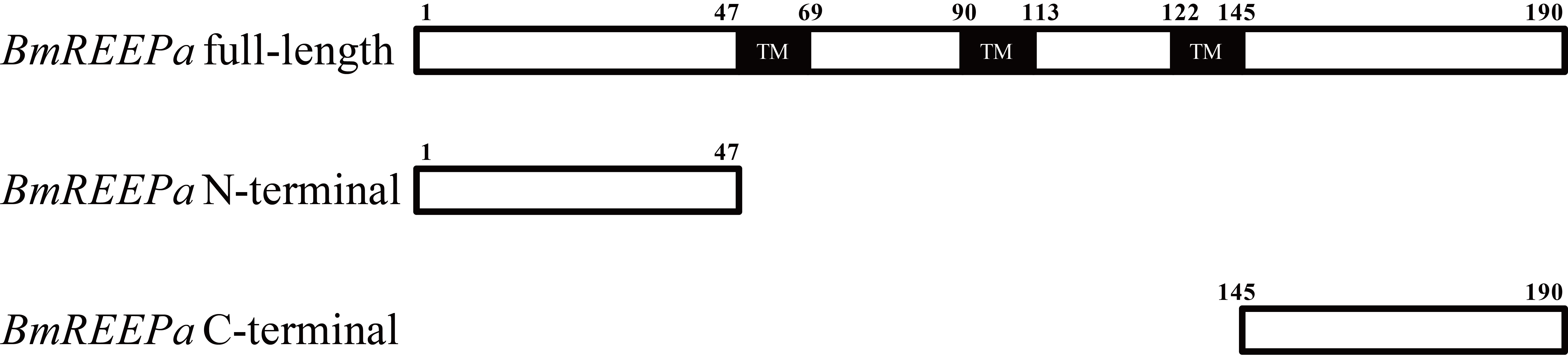

Supplement: S2 Fig — (TIF) [file pone.0144575.s002.tif]
